# Translating a Culture of Quality to Clinical Research Conduct: Expanding the Clinical Development Quality Framework

**DOI:** 10.1007/s43441-023-00610-5

**Published:** 2024-02-07

**Authors:** Michael Torok, Leslie Sam, Jennifer Hebert

**Affiliations:** 1 Global Head of Quality Assurance Programs at Roche/Genentech, 1 DNA Way, Suite 258A, South San Francisco, CA 94080 USA; 2 Leslie Sam and Associates, 2316 New Orchard Court, Sun City Center, FL 33573 USA; 3 Roche/Genentech, 1 DNA Way, Suite 258A, South San Francisco, CA 94080 USA

**Keywords:** Culture, Quality, Open Dialogue, Critical Thinking

## Abstract

The International Council on Harmonisation E8 Guidance Revision 1 (ICH E8(R1)) calls for creating a Culture of Quality that “values and rewards critical thinking and open, proactive dialogue about what is critical to quality.” Across the biopharma landscape, clinical sites, sponsors, and service providers are working to translate this far-reaching guideline into working practices. This manuscript deconstructs key elements that comprise the critical thinking and open, proactive Culture of Quality “enablers.” In addition, maturity models are provided so readers can visualize what a Culture of Quality looks like in their clinical research organization. These provide examples of high performing cultures of quality and useful tools for teams or organizations to measure and evolve their respective quality cultures.

## Introduction

Over the last decade, quality risk management practices within the pharmaceutical industry have evolved to focus on quality by design (QbD), risk-based approaches, and issue prevention [1–3]. One of the most influential guidelines driving this focus was the International Council on Harmonisation E6 Guidance Revision 2 (ICH E6(R2)) released in 2016 [4]. In parallel with similarly themed contemporary publications, this guidance provided a solid foundation for the clinical quality risk management practices that are still informing the work of industry-leading collaborations and thought leaders today.

In October of 2021, the release of ICH E8(R1) moved beyond earlier work to promote culture and the behaviors required to sustain it. General Considerations for Clinical Studies Sect. 3.3.1 “Establishing a Culture that Supports Open Dialogue,” describe these cultural considerations as well as critical drivers influencing quality as.

Creating a culture that values and rewards critical thinking and open, proactive dialogue about what is critical to quality for a particular study or development programme, going beyond sole reliance on tools and checklists, is encouraged. Open dialogue can facilitate the development of innovative methods for ensuring quality” [ 5 ].

The release of draft guidance ICH E6(R3) builds upon these themes and provides in-depth information for those seeking to explain and operationalize the ICH E6 and E8 requirements. Further, International Council on Harmonisation E6 Guidance Revision 3 (ICH E6(R3)) reinforced concepts introduced in ICH E8(R1), by fostering the idea of proactively building quality into trial design starting in the early planning stages and spanning both operational and quality groups.

The guidance of health authorities and published conceptual frameworks provided the initial impetus for individuals and teams to begin implementation of quality risk management practices. [6–16] However, most of these efforts do not fully consider the cultural impact of quality risk management when deploying these frameworks creating a missed opportunity to optimize these practices. Further, this may even result in incomplete adoption by teams despite significant upfront investment of time and effort. In clinical development, there is extensive work required to create realistic translations of conceptual methodologies within the complexity of everyday operations. Therefore, it is not surprising when we sometimes forget that it is *PEOPLE* who design quality into investigational plans and apply risk-based methodologies to clinical development programs. It is *PEOPLE* who execute day-to-day clinical quality management system activities. It is *PEOPLE* whose everyday behaviors enhance or detract from the value of the clinical quality management enterprise. Safe and effective therapeutics are reliant on quality and quality is contingent on *PEOPLE*. Because of this interdependency, the culture of an organization will not only influence the success of quality risk management but also the eventual outcomes of clinical development programs. [17]

ICH E8(R1) requires quality be managed through a Culture of Quality with human behaviors such as Critical Thinking and Open Dialogue at its epicenter. This paper translates the regulatory expectation of a Culture of Quality into meaningful practices for clinical development that will underpin the operational aspects of ICH E6(R3).

## Expanded Culture of Quality Strategic Enablers

**Figure 1:**
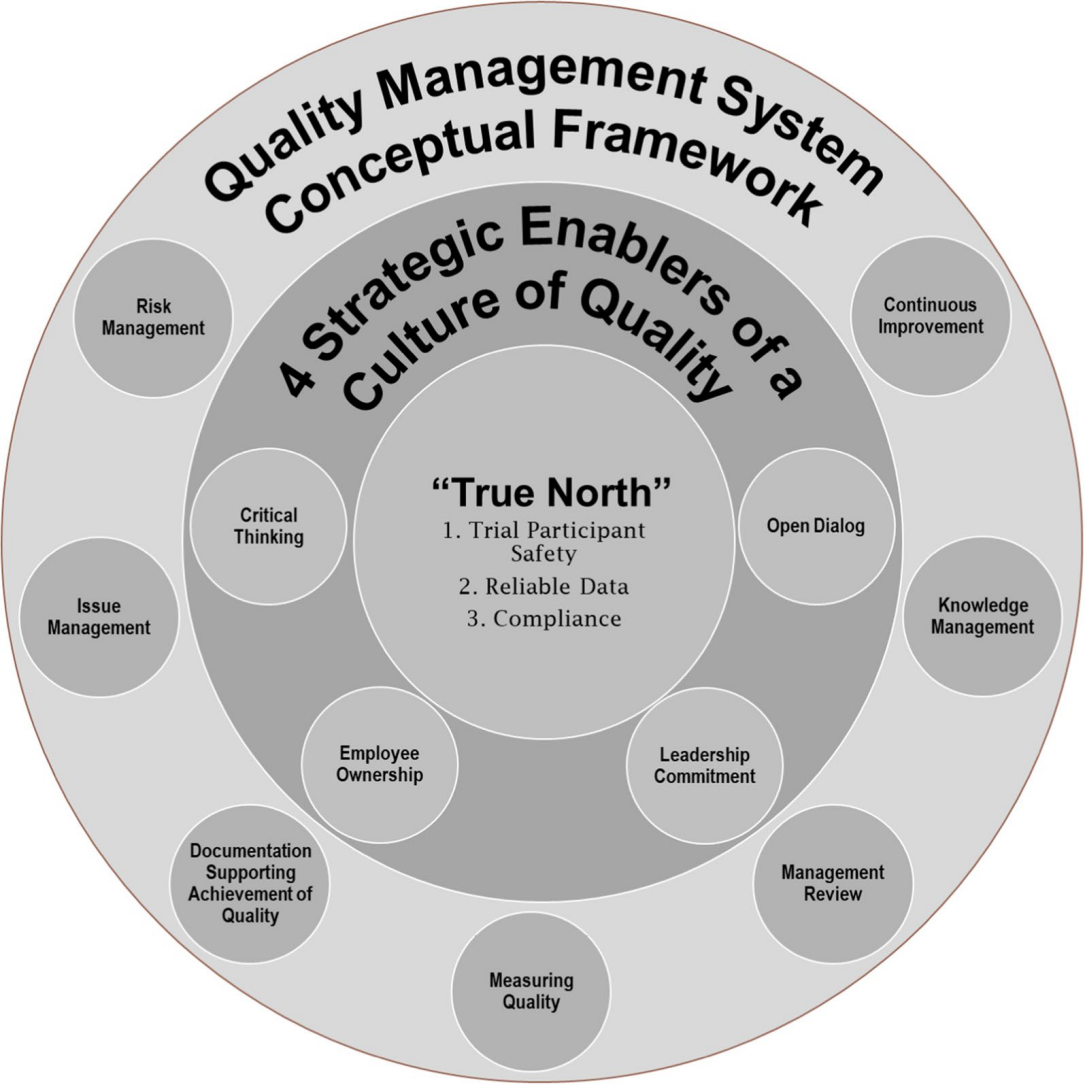
The Culture of Quality Strategic Enablers is the missing piece between the Quality Management System Conceptual Framework and “True North.”

In clinical research, the goals of trial participant protection and reliability of trial results are universally accepted as paramount to successful study conduct. In addition, while these clinical quality objectives are no doubt considered in the initial creation of organizational credos, mission statements, and quality policies, it is important to connect the lines between our overarching direction, our “True North,” and the conceptual Quality Management System (QMS) frameworks employed to govern daily operations. These frameworks include risk and issue management, measuring quality, management review, knowledge management, continuous improvement, and robust documentation to demonstrate the achievement of quality. They build on the foundations of regulatory requirements and thought leadership, lighting the path as we seek to define effective operational strategies. Along the way, we find gaps in quality, failures in oversight and poor outcomes that prove we have not fully identified or optimized these strategies. Moreover, while we can see where we want to go, we are missing the bridge between these conceptual QMS frameworks and our ultimate goals. This is where the utilization of four principal Culture of Quality Strategic Enablers provides a conduit to facilitate our journey to the other side (Fig. 1).

Each of the four Strategic Enablers comprises multiple behaviors and mindsets:

**Table:** 

Strategic enabler	Behaviors and mindsets
Leadership commitment	A committed leadership will support ● Creation of detailed quality goal development ● Aligning rewards and recognition to culture ● Clear accountability for quality performance
Employee ownership	Employees will ● Develop and maintain quality mindsets and values ● Partner with Quality Assurance (QA) to foster an Open Dialogue and seek opportunities to improve Critical Thinking skills ● Make certain that there is documentation to support the achievement of quality objectives
Open dialogue	Open Dialogue is facilitated by ● Accessing and exchanging data and information ● Leveraging diverse intellectual capital ● Quality Partnership and Team Engagement
Critical thinking	Critical Thinking is ● A learned competency taught through application of a systematic approach such as the “4As”: Ask, Analyze, Answer, Act ● Relies on enhanced knowledge and expertise ● Is developed through practice and offers opportunities for continuous improvement

The Open Dialogue and Critical Thinking Strategic Enablers are specifically called out in ICH E8(R1), while the remaining two (Leadership Commitment and Employee Ownership) are considered paramount for driving organizational dynamics to enable success. In this paper, we will describe and provide in-depth operational guidance for the realization of the first two (Open Dialogue and Critical Thinking) identified principal Culture of Quality Strategic Enablers.

## Open Dialogue and Critical Thinking

**Figure 2:**
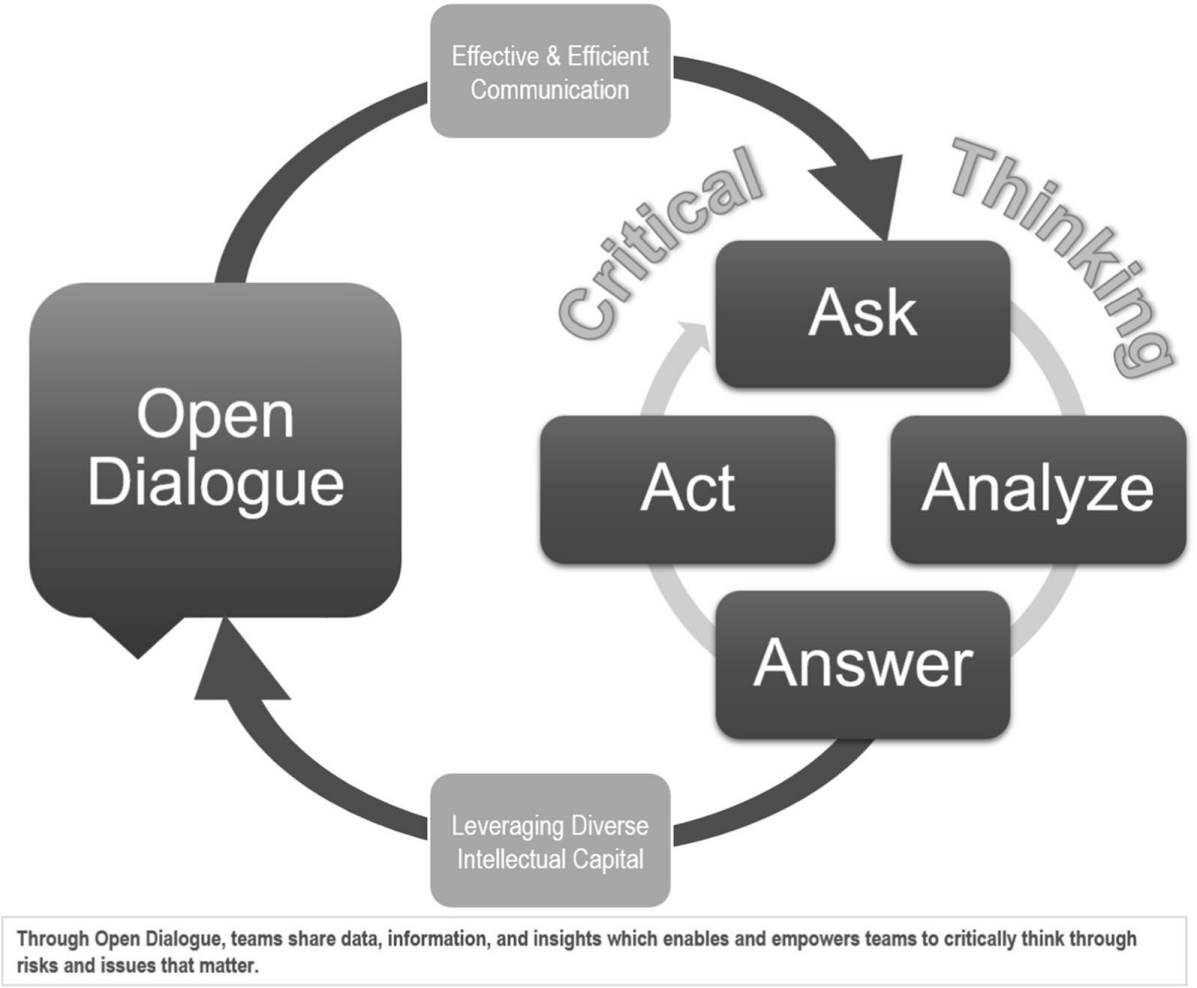
Relationship between Open Dialogue and Critical Thinking Strategic Enablers.

It is important to recognize that Open Dialogue and Critical Thinking work together in a complementary fashion despite being independent concepts. A high performing Culture of Quality consistently demonstrates both Open Dialogue and Critical Thinking in tandem (Fig. 2).

For example, if we expect study teams to think critically about what to include in protocol amendments, it is imperative to provide access to the data and information relevant to the proposed changes as well as support proper analysis of feasibility and operationalization. If the development team fails to include the appropriate subject matter experts (e.g., medical, biostatistics, and quality—some of the intellectual capital for the study), to analyze information through their unique functional lens, there is a high risk of incomplete assessment(s) or poorly considered actions that will not fully address the needs of the study.

## Open Dialogue: Data Access & Exchange

Data access and exchange is a critical factor leading to team success when implementing the Open Dialogue enabler. Poorly designed or overly complicated systems will prevent data from being viewed, openly shared, discussed, or examined, all of which will impair overall quality management practices (e.g., risk management). Such obstacles will frustrate even the most motivated teams seeking to evaluate the risk landscape when they find the systems managing data or creating analytics are too burdensome to use. Moreover, even with an intuitive user interface, a system that does not refresh at the cadence required to provide contemporaneous data creates a risk of inaccurate conclusions or failed analysis. In situations where a serious quality issue or serious breach (SB) occurs, an event may go unnoticed by the development team, and therefore, remain unmitigated, and unreported. This lack of information sharing across functions may leave time-sensitive activities such as SB expedited reporting at significant risk, leading to serious consequences downstream with health authorities.

Organizations with mature Cultures of Quality have user-friendly, quality performance systems, and analytics. Minimally, these mature organizations solve challenges around data interoperability to systematically produce and provide metrics across clinical development departments, vendor data, and QMS data to assess holistically the health of the clinical enterprise, identify process fragility, and predict quality outcomes. Further use cases for individual analysis and support using machine learning (ML)/Artificial Intelligence (AI) also exist; however, any use of data will always rely on the normalization of disparate data sets and the accuracy of underlying data. This leads to the important consideration and the imperative need to standardize data in the clinical development ecosystem. Given the volume of datasets, organizations should strive to have effective data governance models. Such models facilitate the linking and normalization of data so parties can discuss aligned data points while understanding inherent relationships between them.

The right systems and analytics should provide easy-to-mine regulatory, inspection, and quality intelligence. Examples of such critical systems components include:

- A regulatory intelligence system supports and manages effective and draft regulatory requirements.
- An inspection intelligence system is a database of inspection outcomes (how health authorities are interpreting regulations and measuring sponsor performance based on that interpretation).
- A quality intelligence system is a database of organizational QA and compliance data, metrics, analytics, and dashboards.

Overall, teams have greater opportunities for Open Dialogue, Critical Thinking, and proper documentation of both risks and mitigations when they have composite information from systems and intelligence databases. In other words, the capability to distill inspection, regulatory, and quality intelligence into usable inputs enables organizations to successfully metabolize knowledge and implement it in their day-to-day actions. If organizations do not democratize data through user-friendly systems, teams lack the appropriate perspective to manage quality according to the standards set forth by regulatory guidance.

## Open Dialogue: Leverage Diverse Intellectual Capital

The second critical factor driving the value of Open Dialogue is the ability to have the right data, analytics, and knowledge management capabilities available to the right team members. Furthermore, these individuals must demonstrate competency in analyzing data or leveraging regulatory, inspection, and quality intelligence, to be effective when managing quality (e.g., quality risks and issues). Mature teams will continuously work toward mastery of these skills and learn from each other as well as subject matter experts when additional knowledge is required. As some experts will sit outside the immediate team’s department or even organization, effective (e.g., mature) risk management teams must recognize when it is necessary to obtain input from experts when making quality management decisions and when building quality into investigational plan design.

Transparency is the mother of mitigation. Teams need support to get comfortable working through risks and issues across all functions and levels of an organization. Similarly, they need to collaborate proactively with outside stakeholder networks, working across vendors (e.g., CROs), and development partners. To be successful in utilizing Open Dialogue as an enabler, teams must leverage the diverse intellectual capital all around them, in a 360° manner. Access to the right data is important; however, it is equally integral to share the right data, right knowledge, and right information at the right time to the right audience.

## Open Dialogue: Quality Partnership and Team Engagement

Open Dialogue is much more than just communicating in team settings; early discussions will need to include quality team members (e.g., risk and issue management) to keep the flow of the discussion moving in the direction of “True North” where focus re-centers on trial participant protection and reliability of trial results. Quality stakeholders can help promote regular discussions around trial participant safety, data reliability, compliance risks and issues that may otherwise be de-prioritized. An authoritative or “policing” quality presence should be avoided in favor of transparency and partnership. Open lines of communication where study team members feel comfortable asking questions or highlighting concerns enable proactive quality engagement rather than teams waiting until an audit or inspection. Early collaboration in quality discussions enabled by impact analyses and an appropriate understanding of risk-proportionate data and processes is transformative when developing innovative mitigation strategies. During this process, the quality team must build mutual trust and maintain individual integrity during tough discussions, even with change-resistant team members. They must find equilibrium while challenging ideas, not individuals.

Team member engagement is fundamental to effective communication. How can you manage quality if team members are not engaged enough to proactively identify risks and to critically think of a proper control? Effective knowledge management strategies beyond team and 1:1 meetings should be considered when working to develop a high performing Culture of Quality inside an organization. Potential strategies such as podcasts, interactive polling, and pre-recorded messages coupled with breakout sessions and workshops are often effective to encourage meaningful engagement. Mature teams will have forums where individuals in diverse roles will regularly discuss quality matters and learn from one another. Examples of these forums may include:

- Communities of Practice designed to share examples of effective risk controls
- Lessons Learned sessions held after an audit, inspection, or management review of quality and compliance metrics
- Risk Strategy hubs to enable knowledge sharing, questions, concerns, and best practices

Since Open Dialogue is an important Culture of Quality Enabler, organizations must actively exchange data, information, and intelligence to enable Critical Thinking about how best to manage quality. However, organizations who have not mastered all or even some of these activities can still develop the attributes and practices necessary for a high performing Culture of Quality. In this case, a maturity model can be employed to determine an organization’s baseline and identify actions for strengthening the organization’s Culture of Quality.

## Leveraging a Maturity Model to Strengthen Your Culture of Quality

Within the pharmaceutical industry, clinical development has often modeled best practices after Good Manufacturing Practice (GMP). For example, GMP colleagues have led the way by assessing Cultures of Quality and applying GMP rules-based QMS to the judgment-based environment of Good Clinical Practice (GCP). By the mid-2010s, the pharmaceutical GMP industry-leaders had deployed Culture of Quality assessment tools and maturity models, including the 2015 Parenteral Drug Association’s (PDA) Quality Culture Guided Assessment Tool [18] and the 2017 International Society for Pharmaceutical Engineering (ISPE) Cultural Excellence Report. [19]

**Figure 3:**
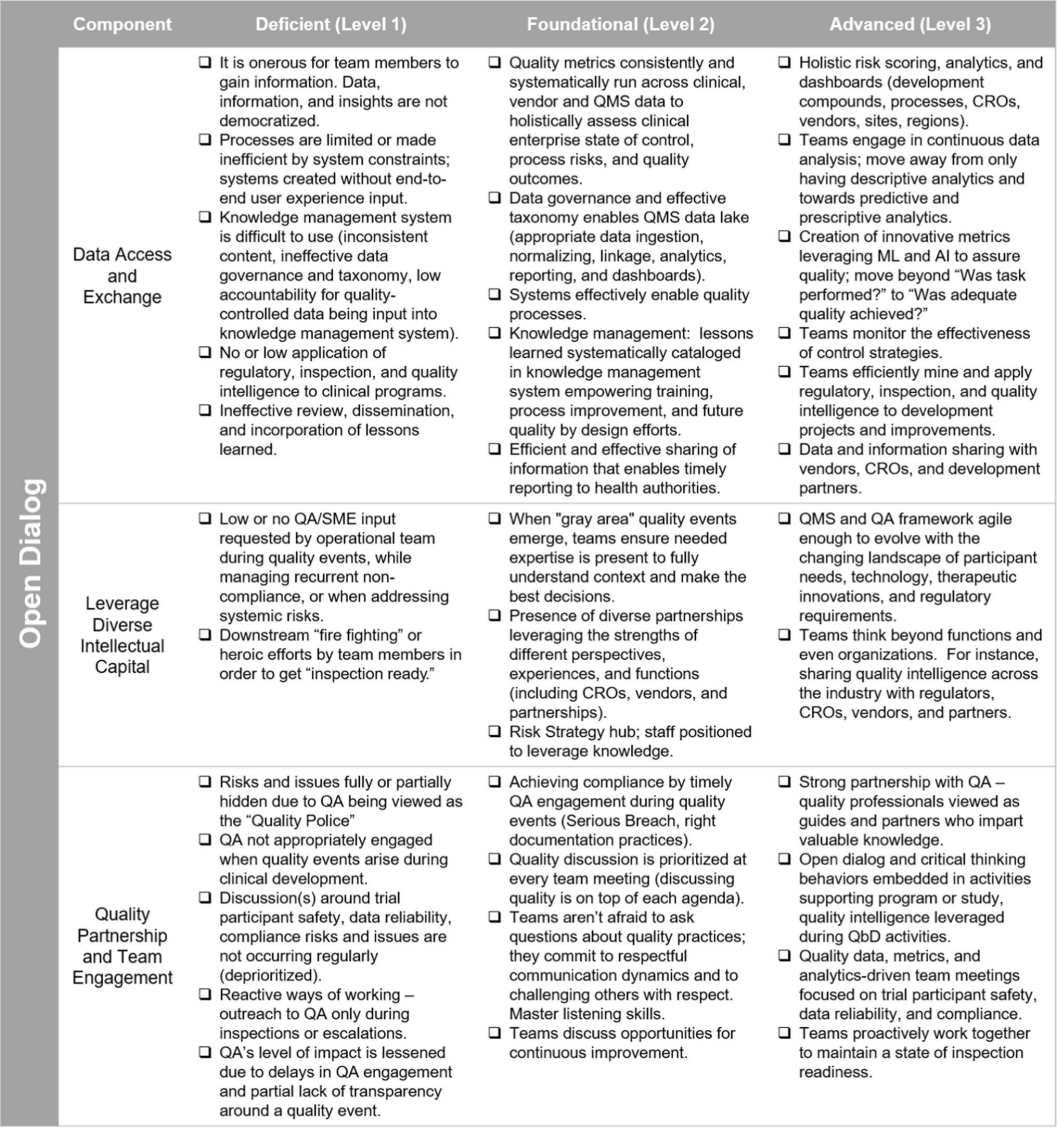
Culture of Quality Strategic Enablers, Open Dialogue Maturity Model.

To facilitate the translation of what a Culture of Quality means for clinical development in the current regulatory environment, we have created assessment tools/maturity models (hereafter maturity models) for the Culture of Quality Strategic Enablers. These maturity models use a self-assessment technique to determine a baseline for the components of the selected Strategic Enabler of current focus. This enables organizations to determine the actions necessary to advance to a higher level of quality performance for each enabler. Long-term, maturity models also provide benchmarking for growth and progress reporting (Fig. 3).

## Critical Thinking: The Critical Thinking Cycle

Study teams face increasingly complex therapeutic areas involving biometrics, genomics, non-traditional study designs, dozens of vendors, disparate internal/external data sources, international site locations, and participant engagement strategies—all while seeking to work more efficiently and achieve a higher standard of quality. In the face of such headwinds, teams will often fall back on reactionary approaches to operational planning and robust quality management (e.g., good documentation, risk management, etc.) is forgotten until a quality issue or inspection arises. Even when teams are able to conduct risk assessments during the start-up phase, as soon as the data (scientific and quality) comes flooding in, team members become overwhelmed and distracted by an endless stream of urgent operational demands. Not surprisingly, proactive Critical Thinking skills tend to atrophy when firefighting becomes the normal way of working. Fortunately, Critical Thinking skills emerge when we strengthen our “mind muscles” using a systematic approach such as the 4 ‘A’s. The core components of this Critical Thinking methodology, the 4 As, are as follows:

- **Asking** questions proactively
- **Analyzing** the data related to those questions
- **Answering** the question
- **Acting** upon the answers

This Critical Thinking practice is action-oriented and should always end with meaningful activity focused on the priorities previously defined. Upon the completion of such action(s), the cycle should begin anew with questions to explore new issues or follow-up on the effectiveness of prior actions. Through vigilance, new questions arise, and drive an examination to determine whether the current information available requires a refresh or expansion to answer the new question.

Critical Thinking in the clinical development arena must also ascertain if the information under review indicates that trial participant protection, reliability of trial results or compliance are now at risk. “Is our ‘True North’ affected?” is the first stop in quality Critical Thinking. To answer this question, teams must allow time for the Critical Thinking cycle to produce well-considered answers even if multiple cycles are required. In order to arrive at the best answers and next steps, the products of Open Dialogue must fuel the Critical Thinking cycle: data acquisition; input from competent subject experts; and effective discussion.

## Leveraging a Maturity Model to Understand and Strengthen Your Culture of Quality and the Critical Thinking Strategic Enabler

**Figure 4:**
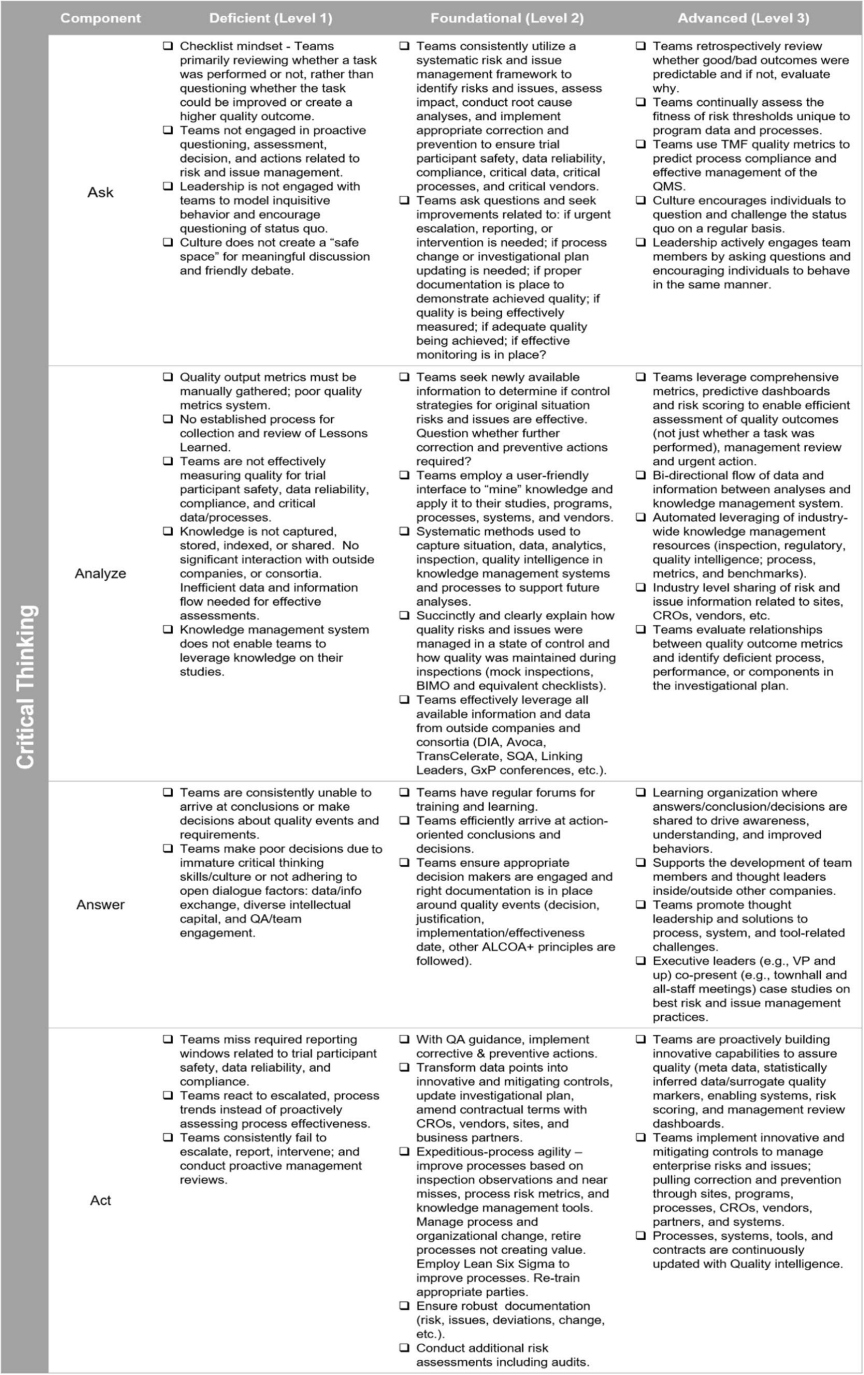
Culture of Quality Strategic Enablers, Critical Thinking Maturity Model.

Similar to the Open Dialogue Strategic Enabler, this maturity model is a self-evaluation tool that can determine an organization’s maturity baseline as well as identify the necessary actions to upskill Critical Thinking within the culture of an organization. During this evolution of culture, even with the support of Quality Enabler maturity models, there will naturally be a need for some degree of change management. Organizations may start as “Deficient” during self-assessment; however, this first baseline provides both an impetus for change and future encouragement to continue as it measures progress when viewed retrospectively. It is expected that functional organizations will have varying levels of maturity, competency, and capabilities but should all be collaborating to enhance its culture of quality (Fig. 4).

Following an initial assessment using the maturity model(s), deficiencies will need to be addressed and associated competencies developed prior to employing the Ask, Analyze, Answer, Act approach. Otherwise, management will need to be prepared to support teams and bridge identified gaps. Once established, as new information becomes available, there are further considerations and questions that can be generated to guide teams initiating the Critical Thinking cycle for the first time. Example questions are found in Appendix A. These questions are intended to provide support and inspire further inquiry but should not be used as an exhaustive list. Each of these questions requires deliberation, consideration, and time to obtain relevant information as per the Critical Thinking model. As teams work through these questions, they should have a curious mindset while staying focused on “True North” to avoid becoming overwhelmed by the data, details, or their own internal biases. In the end, teams may also need to partner with quality and/or regulatory professionals to confirm the appropriateness of agreed action(s). Examples of potential actions are provided in Appendix B. It is important to note that while many of the example questions and actions seem relevant during the execution of investigational plans, most should first be applied during the creation of the investigational plan. QbD principles, ICH E6(R2)/Draft ICH E6(R3) clinical quality management frameworks, and the Culture of Quality Strategic Enablers are of greatest value before a clinical study or program begins.

## Final Thoughts

The Culture of Quality Strategic Enablers brings to life the requirements in ICH E6(R2), *Draft* ICH E6(R3), and the clinical quality management system frameworks. Together, these Culture of Quality enablers Open Dialogue, Critical Thinking, Leadership Commitment, and Employee Ownership expand the Clinical Development Quality Management framework to support operational implementation. This gives clinical development teams a roadmap for cultural growth that will better safeguard trial participant protection, reliability of trial results, and quality compliance. Beyond clinical development, the Culture of Quality Strategic Enablers provides value for a variety of stakeholders spanning sponsor, services, and vendors within our industry. In the end, all contributors to a trial should work together to develop strategies that foster critical thinking and open proactive dialogue.

The more mature an organization’s Culture of Quality, the more agile its navigation of the ever-changing landscape of participant considerations, scientific breakthroughs, innovative therapeutics, and emerging regulatory requirements. Full adoption of these principles will pay dividends as sponsors rededicate resources to clinical trial execution rather than firefighting and re-work. Efficiencies and smarter resource deployment lead to expedited delivery of new therapeutics as well as a happier workforce. These benefits have become crucial to the future state of organizations such that individuals should not wait for leadership directives but instead take the initiative to begin exhibiting behaviors in line with these Strategic Enablers. The work should begin in earnest with the germination of a Culture of Quality at every level and function. We are all on a journey together and together we will reach our “True North.”

## Data Availability

Not applicable.

## Appendix A

Examples of Questions to Initiate the Critical Thinking Cycle.

- As I execute this investigational plan, does this new information affect trial participant safety, data reliability, or compliance?
- Does this information affect the ability to conclude the study?
- If the right data is not available to me, what surrogate data might help me critically evaluate this information?
- Given this information, is urgent escalation, reporting, or intervention needed?
- What is the root cause of the related risk or issue?
- Did the team know this quality event was going to happen – if not, why?
- What is the appropriate correction/remediation given this information?
- What risks will the team accept?
- For this situation, has the QMS been appropriately used?
- Has the team robustly documented (followed ALCOA +) this situation in the TMF?
- During an inspection, can I explain how I maintained a state of control while managing this risk or issue?
- Given this information, do I need to modify the control strategies or investigational plan?
- How will the effectiveness of the new control strategy be measured?
- What additional quality performance measures or monitoring do we need to protect trial participant safety, data reliability, or compliance?
- How does this information broadly affect the clinical enterprise including other programs, and enterprise-wide processes, systems, or vendors (impact assessment)?
- Is there a systemic issue that needs to be addressed?
- Is process change needed?
- Have I helped my organization learn from this information?

## Appendix B

Examples of Potential High-level Actions in the Critical Thinking Cycle

- Report serious quality events (e.g., SB, recurrent non-compliance) to health authorities as appropriate.
- Urgently escalate the situation as appropriate (medical, data integrity department, management review, legal, etc.).
- Implement corrective and preventive actions.
- Transform data into innovative, mitigating controls that are proportional to the risks.
- Implement innovative, mitigating controls.
- Update the investigational plan to prevent future issues.
- Update contractual terms or governance documentation for CROs, vendors, or development partners.
- Retrain associated parties.
- Update related processes.
- Conduct additional risk assessments.
- Appropriately capture quality event information and remediation in knowledge management system to support learning and future improvement.

## Abbreviations

AI: Artificial Intelligence
ALCOA+: Principle that is crucial for ensuring data reliability representing the following principles (Attributable, Legible, Contemporaneous, Original, Accurate). The " + " sign indicates the inclusion of additional principles that further enhance data integrity. While variations exist, the " + " may represent principles such as (Complete, Consistent, Enduring)
AVOCA: The Avoca Quality Consortium, a WCG company—A consulting and research firm in the life sciences industry, specializing in clinical research and quality improvement
BIMO: Bioresearch Monitoring Program
CROs: Contract Research Organizations
CTTI: Clinical Trials Transformation Initiative
DIA: Drug Information Association
GCP: Good Clinical Practice
GLP: Good Laboratory Practice
GMP: Good Manufacturing Practice
GxP: Collective term used to refer to various Good Practice guidelines, with the "G" representing "Good" and the "P" standing for "Practice." The "x" is a placeholder for the specific letter(s) implied depending on the context. Common examples: GCP, GLP, GMP
ICH E6(R2): International Council on Harmonisation E6 Guidance Revision 2
ICH E6(R3): International Council on Harmonisation E6 Guidance Revision 3
ICH E8(R1): International Council on Harmonisation E8 Revision 1
ICH: International Council on Harmonisation
ISPE: International Society for Pharmaceutical Engineering
ML: Machine Learning
PDA: Parenteral Drug Association
QA: Quality Assurance
QbD: Quality by Design
QMS: Quality Management System
SB: Serious Breach
SME: Subject Matter Expert
SQA: Society of Quality Assurance
TMF: Trial Master File
